# The Pew Nuisance

**Published:** 1872-09

**Authors:** 


### THE PEW NUISANCE.

No wonder there is a clamor for opening libraries, and reading-rooms, and picture galleries on Sundays. It is a fact that our young men had better go to these than spend their time at corrupting and beer-drinking Sunday concerts, in idle lounging in the Central Park, or about the doors of cigar shops, and at candy counters. But as for going to church, they have no church to go to. A single sitting in a respectable place of worship, costs from twenty-five to fifty dollars a year, and very few of our clerks and apprentices can spare that much, or anything like it, especially as many of them have parents, or sisters, or other relatives who look to them for some help. And even on those occasions when the churches are advertised in the daily papers as being opened to the public for a special purpose, — that is, to draw a crowd, and after the performances to hand around a broad silver plate, to compel you to put in a spare nickel, — if you do happen to drop in, you don't know where to go, and have to stand until all patience and temper is gone, with your finger in your mouth, waiting for some man to invite you to a seat — but he don't; and if you invite yourself to one, you stand an even chance of being bowed out.

Very recently a grand building on Fifth Avenue was advertised to be thrown open to the public, to hear a distinguished Italian orator. We accepted the invitation for ourself and a lady. Went early. There was plenty of room; so we selected the best place, as we always do when we have a chance — and why not? In little or no time a prim maiden person sidled up to us, and with the blandness which only an

"AH SIN"

could equal, volunteered the information that "All our family will be here to-night!" As we were saying, this is the beginning of the end of the nineteenth century in Fifth Avenue, New York city. The assertion may be ventured that we are not the only person who has been invited out of a pew, instead of into one, in this great centre of civilization. The truth is, we ought not to have gone to that pew, for we have three standing invitations to sittings in the centre aisle, but we thought there was to be a free fight, and everybody was invited to come by the distinguished minister who officiates habitually; but his parishioners do not seem to care that everybody should come to their pews; they no doubt would be very willing for crowds to come, if they only occupied somebody else's pew.

